# Effect of subsequent vaginal delivery on bowel symptoms and anorectal function in women who sustained a previous obstetric anal sphincter injury

**DOI:** 10.1007/s00192-018-3601-y

**Published:** 2018-03-29

**Authors:** Polly A. Jordan, Madhu Naidu, Ranee Thakar, Abdul H. Sultan

**Affiliations:** 1 Urogynaecology Unit, Croydon University Hospital, 530 London Road, Croydon, CR7 7YE UK; 2 St. George’s University of London, London, UK

**Keywords:** Obstetric anal sphincter injuries, Subsequent pregnancy, Third-degree tears, Anal sphincter, Childbirth, Vaginal delivery

## Abstract

**Introduction and hypothesis:**

Our primary objective was to prospectively evaluate anorectal symptoms, anal manometry and endoanal ultrasound (EAUS) in women who followed the recommended mode of subsequent delivery following index obstetric anal sphincter injuries (OASIs) using our unit’s standardised protocol. Our secondary objectives were to evaluate the role of internal anal sphincter defects and also to compare outcomes in a subgroup of symptomatic women with normal anorectal physiology.

**Methods:**

This is a prospective follow-up study of pregnant women with previous OASIs who were counselled regarding subsequent mode of delivery between January 2003 and December 2014. Assessment involved the St Mark’s Incontinence Score (SMIS), anal manometry and EAUS at both antepartum and 3-month postpartum visits. Data were analysed using Wilcoxon and Mann–Whitney *U* tests.

**Results:**

Three hundred and fifty women attended the perineal clinic over the study period, of whom 122 met the inclusion criteria (99 vaginal delivery [VD], 23 caesarean section). No significant worsening of anorectal symptoms was observed following subsequent delivery in the VD group (*p* = 0.896), although a reduced squeeze pressure was observed at 3 months postpartum (*p* < 0.001). There were no new defects on EAUS in either group.

**Conclusions:**

This study showed no significant worsening of bowel symptoms and sphincter integrity apart from lower squeeze pressures at 3 months postpartum in the VD group when our standardised protocol was used to recommend subsequent mode of delivery. In the absence of a randomised study, use of this protocol can aid clinicians in their decision-making.

## Introduction

The reported rate of obstetric anal sphincter injuries (OASIs) is rising and in England alone the rate in primiparous women has trebled over a 10-year period [1]. Consequently, there is a need for a consistent evidence-based approach to managing women who have previously sustained OASIs in a subsequent pregnancy. A Cochrane review in 2014 studying the effectiveness of interventions for women in a subsequent pregnancy found no trials of sufficient scientific rigour to fit the inclusion criteria [2]. Since then, a number of studies have been published that have focused on rates of recurrent OASIs [3, 4], and the association between OASIs and anal incontinence [5, 6]. The Royal College of Obstetricians and Gynaecologists (RCOG) [7] guideline for the management of third- and fourth-degree tears has recently been updated, but it is interesting to note that the advice regarding management of women in a subsequent pregnancy has not changed, which probably reflects the fact that the evidence base has progressed little since the previous publication in 2007 [8]. Small numbers of patients with recurrent OASIs, limited facilities in many centres for performing anorectal physiology testing/anal ultrasound, and ethical issues have restrained rigorous research and implementation of randomised trials.

OASIs are associated with significant physical morbidity, including perineal pain, sexual dysfunction, urinary, and anal incontinence [2]. This burden of physical morbidity can be associated with significant psychological morbidity, although the relationship between physical birth trauma and consequent psychological morbidity remains under-recognised and probably under-treated [9]. Of particular concern following OASIs is the development of anal incontinence, as demonstrated in a recent meta-analysis, which showed a significant association between OASIs and anal incontinence (OR 2.66, 95% CI 1.77–3.98) [5].

A large cohort study showed the rate of recurrent OASIs to be 7.2% for women who had previously sustained OASIs during their first vaginal delivery, compared with a rate of 1.3% for women who did not [3]. Subsequent vaginal delivery is therefore associated with increased risk of sustaining a further OASI. However, whilst the alternative mode of delivery (caesarean section) is protective against new sphincter defects, there is also a risk of associated morbidity [10].

The important clinical question of how to counsel a woman regarding mode of delivery in a subsequent pregnancy following the index OASI remains largely unanswered. Clinicians and women can therefore face a difficult dilemma when the situation arises. In 2009, Scheer et al. [11] were the first to prospectively evaluate women for anorectal symptoms and carried out anorectal manometry, endoanal ultrasound (EAUS) and quality of life (QoL) assessments before and after subsequent childbirth following the index OASI. They demonstrated that in the absence of antenatal evidence of anal sphincter dysfunction, there was no deterioration in anorectal symptoms, manometry or EAUS findings. The study is limited, however, by the relatively small number of participants. Karmarkar et al. [12] published another small study based on the same criteria as that described by Sheer et al. [11] and reported similar findings. However, there is currently no literature reporting postpartum outcomes for symptomatic pregnant women with normal anal manometry and EAUS and this particular group has consequently not been provided with specific guidance in previous published protocols, but rather has formed part of the “equivocal” group.

The primary objective of this study was to compare symptoms of anal incontinence, sphincter function, and sphincter integrity among women who were recommended either vaginal delivery (VD) or caesarean section (CS) as per the standardised protocol. Our secondary objectives were first to establish the influence of concomitant internal sphincter defects on symptoms following VD, and second to compare the same outcomes in a subgroup of symptomatic women with normal EAUS, but either normal or abnormal manometry (incremental squeeze pressure > 20 mmHg) who delivered by VD.

## Materials and methods

### Selection and description of participants

In this prospective cohort study, consecutive pregnant women who had sustained previous OASIs were seen in a subsequent pregnancy both in the antenatal and postnatal period in a specialist perineal clinic between January 2003 and December 2014. A small number of women within this cohort have been included in a previously published study [11]. OASIs were classified according to the Sultan classification [13] adopted by the RCOG [7, 8] and the Joint ICS/IUGA Terminology document [14].

Patients were included if they sustained OASI in a previous delivery, attended the perineal clinic for both antenatal and postnatal appointments and completed all assessments. Mode of delivery was either by VD or elective CS. Patients were excluded if there was no clear documentation or evidence of previous OASIs, if they did not have completed paired data (antenatal + postnatal), and also women who underwent emergency CS for obstetric or fetal reasons.

A subgroup of symptomatic women with normal anorectal manometry and EAUS (incremental pressure > 20 mm of mmHg plus no defect on EAUS) were identified to compare the effect of mode of delivery (VD vs CS) on postpartum outcomes.

Ethical approval was not required as this work was registered with the Audit Department (2015/43) and undertaken as part of an on-going clinical audit process.

### Data collection

Participants underwent comprehensive antenatal assessment between 28 and 32 weeks’ gestation including evaluation of anorectal symptoms [14], anal manometry [15] and EAUS [14, 15]. Symptoms were assessed using the validated St Mark’s Incontinence Score (SMIS), which grades severity of anal incontinence on a scale of 0–24 with 24 being severe incontinence [16]. Anal manometry was performed using a validated Stryker 295 air-filled balloon manometer [15]. The maximum squeeze pressure (MSP), maximum resting pressure (MRP) and anal length were measured. EAUS was performed using the 10–16 MHz 360° rotating probe (BK Medical). Three-dimensional images were then reviewed by practitioners experienced in pelvic floor and anal sphincter ultrasound and were viewed at four levels: puborectalis, deep (proximal), superficial (mid) and subcutaneous (distal) external anal sphincter (EAS).

**Fig. 1:**
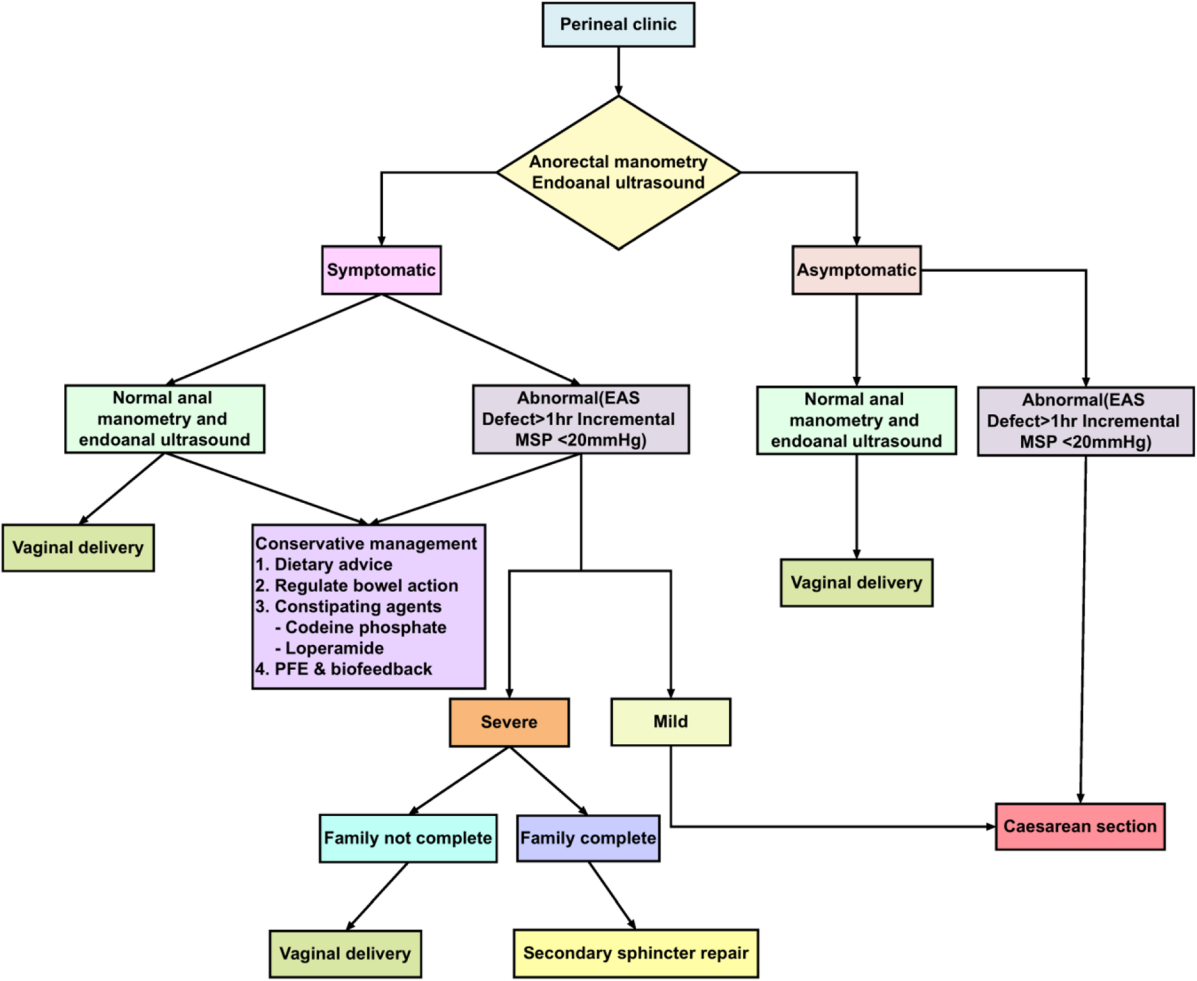
Protocol used to decide on the mode of subsequent delivery in women with a history of obstetric anal sphincter injuries (OASIs)

**Fig. 2:**
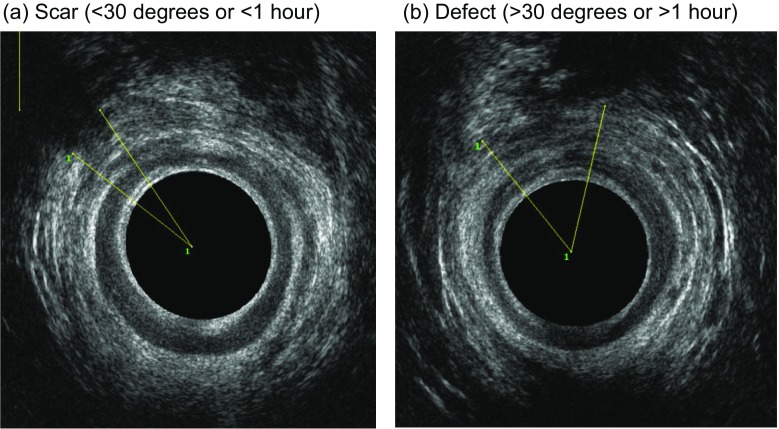
Endoanal ultrasound images illustrating scar versus defect

In our Perineal Clinic we follow a set protocol for the management of women in a subsequent pregnancy and advice was given to women regarding subsequent mode of delivery on the basis of this protocol (Fig. 1). VD was recommended if symptoms were minor (flatus incontinence or occasional passive soiling) or the woman was asymptomatic, and the EAS scar extended for less than 1 h on the clock face (30° angle) and the squeeze incremental pressure was >20 mmHg (Fig. 2a). CS was recommended for all others (Fig. 2b). Patients then underwent the same comprehensive assessment at 8–12 weeks postpartum.

### Data analysis

Demographic and obstetric factors were obtained and the women were divided into two groups according to mode of delivery (VD or CS). Longitudinal comparison of antenatal and postnatal SMIS, anal manometry and EAUS were first compared. Antenatal and postnatal anorectal symptoms, anal manometry and EAUS were then compared between groups.

Statistical analysis was performed using SPSS (v20). Longitudinal data for SMIS score was analysed using the Wilcoxon signed rank test and the groups were compared using the Mann–Whitney *U* test. Paired *t* test was used for longitudinal comparison of anal manometry and independent *t* test was used to compare between the groups. Similar analysis was performed on the subgroup. Results were considered statistically significant if *p* < 0.05.

## Results

### Whole cohort

**Fig. 3:**
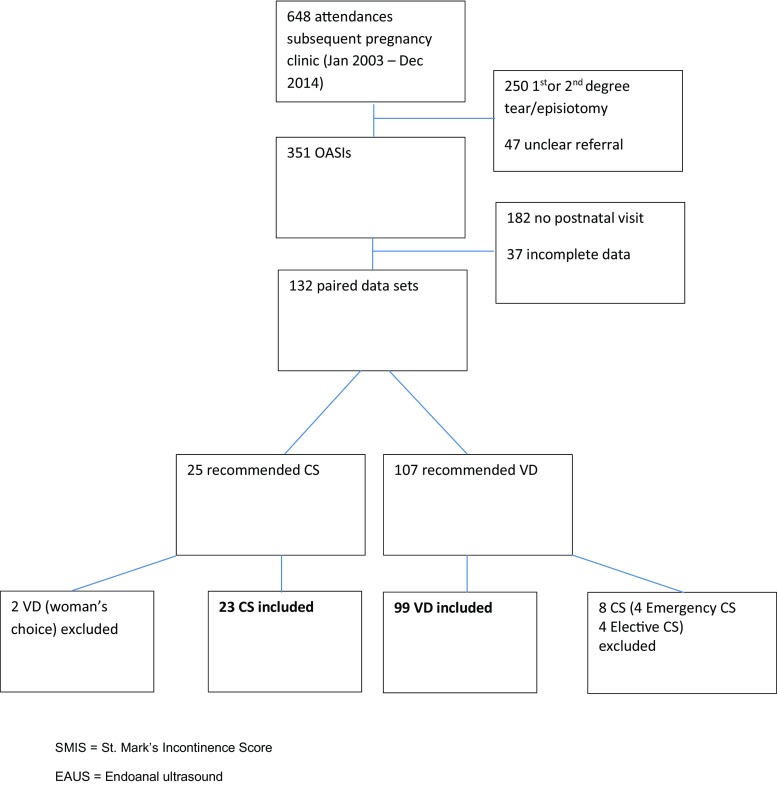
Flow sheet outlining women included in this study

A total of 648 women attended the perineal clinic in a subsequent pregnancy over the study period. Of these, 351 had OASIs and 122 met the strict inclusion criteria (Fig. 3). The predominant ethnic groups were White British (45%), Asian Indian (28%) and Black African (13%). The mean age was 34 years (range: 23–43) and the mean body mass index was 25 (17–42). On the basis of our protocol, 107 (81%) women were recommended for VD. Of these women, 8 were planning to follow our advice, but underwent CS for either elective reasons (*n* = 4) or obstetric reasons (*n* = 4) and were therefore excluded. Twenty-five (19%) women were recommended for CS. Of these, 2 women underwent VD as per their choice and were excluded.

The following rates of OASIs were sustained at index delivery: 26% 3a tear, 30% 3b tear, 13% 3c tear, 7% had a fourth-degree tear; 24% of tears were unclassified. The overall rate of recurrent OASIs was 10% (10 out of 99); 7 (70%) were classified as 3a, 1 (10%) as 3b, 2 (20%) as 3c. There were no new defects on EAUS in this group and no significant change in total SMIS or anal manometry.

When performing a longitudinal comparison of SMIS in antenatal and postnatal women, within each assigned method of delivery group (Table 1), there was no significant change in the total SMIS score following subsequent delivery in the VD group (*p* = 0.896). In the CS group, an improvement in faecal urgency (*p* = 0.026) and quality of life (*p* = 0.011) was observed 3 months postpartum (Table 1). The same analysis for anal manometry outcomes (Table 2) showed that compared with antepartum measurements, squeeze pressure at 3 months postpartum was significantly lower in the VD group (*p* = <0.001) and there was no significant change in the CS group. There were no other significant differences within mode of delivery groups over time.

**Table 1:** Comparison of antenatal and postnatal St Mark’s Incontinence Score (SMIS) within each mode of delivery group

	VD (*n* = 99)				CS (*n* = 23)			
	Better	Worse	No change	*p* value**	Better	Worse	No change	*p* value**
Solid	0	0	84	1.00	1	0	21	0.317
Liquid	0	4	80	0.066	5	1	16	0.111
Flatus	9	7	6	0.834	8	3	11	0.078
Urgency	2	8	74	0.085	6	0	16	0.026
Quality of life	5	3	76	0.394	9	1	12	0.011
Total score, mean (SD)*	Antenatal 0.81 (2.20)	Postnatal 0.84 (2.68)		0.889	Antenatal 3.87 (4.69)	Postnatal 5.83 (5.41)		0.038

No missing values in total SMIS, but there were some missing values in the individual components in 12 women

*SD*, *VD* vaginal delivery, *CS* caesarean section

*Paired *t* test

**Wilcoxon test

**Table 2:** Comparison of antenatal and postnatal anal manometry within each mode of delivery group

	VD (*n* = 99)				CS (*n* = 23)			
	Antenatal	Postnatal	*p* value*		Antenatal	Postnatal	*p* value*	
Anal length	23.76 (5.55)	24.11 (5.99)	0.658		17.70 (6.31)	18.26 (7.47)	0.77	
Resting pressure	48.71 (14.86)	49.57 (15.03)	0.599		30.86 (11.50)	33.5 (15.57)	0.42	
Squeeze pressure	92.02 (30.85)	49.90 (32.28)	0.000		53.86 (16.78)	61.95 19.32)	0.05	

*Paired *t* test

Table 3 shows a comparison of antenatal VD versus antenatal CS, and postnatal VD versus postnatal CS. Before delivery (antenatal), the CS group was significantly worse in terms of flatus and liquid stool incontinence, QoL, anal length, resting and squeeze pressures. These baseline differences between groups are to be expected, as SMIS and anal manometry are both used to guide the stratification of patients regarding mode of delivery. After delivery (postnatal), there was a significant difference in all parameters except squeeze pressures.

**Table 3:** Comparison of St Mark’s Incontinence Score (SMIS) and anal manometry in antenatal VD versus antenatal CS, and postnatal VD versus postnatal CS

	Vaginal delivery (*n* = 99)		Caesarean section (*n* = 23)		*p* value*	
	Antenatal	Postnatal	Antenatal	Postnatal	Antenatal (VD vs CS)	Postnatal (VD vs CS)
SMIS mean score (SD)*						
Solid	0.00 (0.00)	0.04 (0.42)	0.00(0.00)	0.05 (0.21)	1.000	0.996
Liquid	0.09 (0.45)	0.04 (0.42)	0.77 (1.27)	1.18 (1.70)	0.000	0.000
Flatus	0.31 (0.92)	0.35 (0.96)	1.23 (1.66)	1.82 (1.68)	0.001	0.000
Urgency	0.25 (0.81)	0.06 (0.38)	0.32 (1.04)	0.86 (1.32)	0.732	0.000
Quality of life	0.13 (0.63)	0.22 (0.80)	0.77 (1.27)	1.59 (1.76)	0.001	0.000
Total score	0.80 (2.19)	0.84 (2.68)	3.87 (4.69)	5.83 (5.41)	0.000	0.000
Anal manometry mean (SD)*						
Anal length (mm)	23.76 (5.55)	24.11 (5.99)	17.70 (6.31)	18.26 (7.47)	0.000	0.000
Resting pressure (mmHg)	48.71 (14.86)	49.57 (15.03)	31.26 (11.39)	33.50 (15.57)	0.000	0.000
Squeeze pressure (mmHg)	92.02 (30.85)	49.90 (32.28)	54.35 (16.55)	61.95 (19.32)	0.000	0.095

*Independent *t* test

In the VD group (*n* = 99), 16 women had internal anal sphincter (IAS) defects (16%). There was no worsening of symptoms at 3 months postpartum following VD in either the intact IAS group or the defective IAS group (Tables 4, 5). However, there was significant reduction in squeeze pressure (<0.001) in the VD group and an increase in squeeze pressure (<0.001) in the CS group. There was also no change in the maximum resting pressure, which reflects IAS function.

**Table 4:** Internal anal sphincter (IAS) defects and outcomes based on recommendation for VD following the protocol (normal external anal sphincter [EAS])

	IAS intact (*n* = 83)				IAS defect (*n* = 15)			
	Better	Worse	No change	*p* value**	Better	Worse	No change	*p* value**
Solid	0	0	70	1.000	0	0	14	1.000
Liquid	0	2	68	0.180	0	2	12	0.180
Flatus	6	3	61	0.549	3	4	7	0.732
Urgency	1	6	63	0.054	1	2	11	0.593
Quality of life	4	1	65	0.102	1	2	11	0.414
Total score, mean (SD)*	Antenatal 0.63 (1.92)	Postnatal 0.84 (2.83)		0.305	Antenatal 1.80 (3.34)	Postnatal 0.87 (1.80)		0.316

*Paired *t* test

**Wilcoxon test

No missing values in total SMIS, but there were some missing values in the individual components in 12 women

**Table 5:** IAS defects and outcomes based on recommendation for VD following the protocol (manometry)

	IAS intact (*n* = 83)				IAS defect (*n* = 15)			
Mean (SD)	Antenatal	Postnatal	*p* value*	Antenatal	Postnatal		*p* value*	
Anal length	23.70 (5.59)	24.36 (6.14)	0.466	24.33 (5.62)	22.67 (5.30)		0.290	
Resting pressure	49.60 (14.78)	50.72 (15.28)	0.538	45.07 (14.83)	43.67 (12.86)		0.719	
Squeeze pressure	94.33 (31.69)	50.67 (33.01)	<0.001	82.27 (22.39)	44.73 (29.48)		0.001	

*Paired *t* test

No missing values for anal manometry

### Subgroup analysis

The subgroup was defined as symptomatic women (SMIS >0) with no defect on EAUS and who have been advised to undergo VD. Fifteen women from our initial cohort (*n* = 137) met these criteria. Of these 15 women, 9 had normal anorectal physiology (incremental pressure > 20 mmHg) and 6 had abnormal anorectal physiology (incremental pressure < 20 mmHg).

There was no significant difference for all the SMIS subsets (Tables 6, 7) in both the groups. When comparing the groups directly, the only change in significance between groups occurred for postpartum total SMIS score (*p* = 0.007) and antepartum squeeze pressure (*p* = 0.011), which worsened in the abnormal anorectal physiology group (Table 8). There were no new defects on EAUS in either group. However, there were four women with IAS defects in the normal physiology group versus one woman in the abnormal physiology group. Two women had worsening of the defects in the normal physiology group versus woman in the abnormal physiology group.

**Table 6:** Subgroup analysis: longitudinal comparison of SMIS in normal versus abnormal anorectal physiology among symptomatic women with normal endoanal scans who were recommended to undergo VD

	Normal physiology (*n* = 9)				Abnormal physiology (*n* = 6)			
	Better	Worse	No change	*p* value**	Better	Worse	No change	*p* value**
Solid	0	0	9	1.000	0	0	3	1.00
Liquid	0	3	6	0.109	0	0	3	1.00
Flatus	2	4	3	0.245	0	1	2	0.31
Urgency	0	6	3	0.024	0	2	4	0.18
QoL	1	1	7	0.655	0	1	2	0.31
Total score, Mean, SD*	Antenatal 4.44 (2.60)	Postnatal 1.11 (2.26)		0.762	Antenatal 13 (1.41)	Postnatal 14 (2.82)		0.434

*Paired *t* test

**Wilcoxon test

No missing data for SMIS

**Table 7:** Subgroup analysis: longitudinal comparison of anal manometry in the normal versus abnormal anorectal physiology among symptomatic women with normal endoanal scans who were recommended to undergo VD

	Normal physiology (*n* = 9)				Abnormal physiology (*n* = 6)			
Mean	Antenatal	Postnatal	*p* value*		Antenatal	Postnatal	*p* value*	
Anal length	26.11 (7.40)	25 (5.59)	0.430		23.33 (2.58)	20.83 (3.76)	0.203	
Resting pressure	51 (18.68)	56.78 (17.61)	0.572		44 (13.74)	40.50 (9.20)	0.341	
Squeeze pressure	90.67 (25.49)	43.44 (32.57)	0.217		56.50 (13.78)	57.33 (15.53)	0.900	

No missing data anal manometry

*Paired *t* test

**Table 8:** Subgroup analysis: comparison of SMIS and anal manometry in normal versus abnormal anorectal physiology among symptomatic women with normal endoanal scans who were recommended to undergo VD

	Normal physiology (*n* = 9)		Abnormal physiology (*n* = 6)		*p* value**	
	Antenatal	Postnatal	Antenatal	Postnatal	Antenatal	Postnatal
SMIS						
Solid	6.5	6.5	6.5	8.13	1.000	0.503
Liquid	7.0	6.5	5.0	8.13	0.482	0.503
Flatus	6.22	6.06	7.33	9.13	0.727	0.199
Urgency	6.33	7.00	7.00	7.00	0.864	1.000
QoL	5.61	5.61	9.17	10.13	0.145	0.050
Total score, mean (SD)*	4.44 (2.63)	1.11 (2.26)	6.50 (3.20)	8.50 (6.38)	0.195	0.007
Anal manometry						
Mean (SD)						
Anal length	26.11 (7.40)	25 (5.59)	23.33 (2.58)	20.83 (3.76)	0.398*	0.136*
Resting pressure	51 (18.68)	56.78 (17.61)	44 (13.74)	40.50 (9.20)	0.448*	0.059*
Squeeze pressure	90.67 (25.49)	43.44 (32.57)	56.50 (13.78)	57.33 (15.53)	0.011*	0.352*

*Independent *t* test

**Mann–Whitney *U* test

## Discussion

In this prospective study of women who sustained OASIs, we found no significant worsening of anorectal symptoms, sphincter function and sphincter integrity when recommendation for subsequent mode of delivery was based upon a comprehensive antenatal assessment comprising SMIS, anal manometry and EAUS. When analysing sphincter function, a significantly lower postpartum squeeze pressure was noted in the VD group, a trend that was also observed in the subgroup of symptomatic women with normal anorectal physiology. This is perhaps an unsurprising finding in the immediate postpartum period following VD and may be a transient state that is part of normal postpartum physiology. Indeed, a previous study assessing the effect of pregnancy and childbirth on pelvic floor muscle function reported worsening squeeze pressures at 14 weeks postpartum, which had recovered completely by 1 year postpartum [17].

Although overall anorectal symptoms for the whole cohort remained unchanged following VD and CS, improvements in the CS group were noted in quality of life (*p* = 0.011) and symptoms of faecal urgency (*p* = 0.026) at 3 months postpartum. This is likely to be attributable to the effect of pregnancy exacerbating symptoms of faecal incontinence in women with established symptoms, with a return to baseline following delivery. An increase in urgency symptoms was also seen in the VD subgroup, and it is perhaps unsurprising that women who were symptomatic before delivery may experience some worsening of symptoms in the immediate postpartum period, a phenomenon that is likely to recover with time as can be seen with squeeze pressure [17]. A study with greater numbers and longer-term follow-up is needed to be able to comment on the significance of this finding, but our initial results are encouraging and suggest that if our triage criteria are followed, symptomatic women with normal physiology could have a VD with good anorectal outcomes, enabling the morbidity burden from CS to be reduced.

To the best of our knowledge, this study is the largest cohort study assessing bowel symptoms, sphincter integrity and anorectal function among women who underwent a subsequent delivery following index OASIs. We are only aware of only two other studies that have used bowel symptoms, anorectal function and sphincter integrity in both their antenatal assessment to recommend mode of delivery using a structured protocol, and then in the postnatal assessment for the evaluation of outcomes [11, 12]. These studies included 59 and 50 women respectively [11, 12]. Both of these studies showed no worsening of bowel symptoms or anorectal physiology when women were appropriately selected for VD following OASI using comprehensive assessment methods. Their results are consistent with our findings. A key difference in our protocol for this study and indeed the RCOG guidelines and all other published work is the advice given to symptomatic women with normal anorectal physiology [7, 12]. According to our protocol, symptomatic women with normal anorectal physiology, defined as an incremental pressure of >20 mmHg and no defect on EAUS, may be advised to have a VD.

Although in-depth antenatal and postnatal assessment is important, equally important is how this information is then used to counsel women regarding mode of delivery. Fitzpatrick et al. recently published a study that used validated symptom scores, EAUS and manometry in the antenatal period to help inform decision-making with regard to subsequent mode of delivery [18]. The criteria used enabled antenatal anorectal physiology results and symptom scores to suggest which women should have a VD versus CS in clear cut cases. The use of this particular set of criteria, however, left a relatively large number of “equivocal” cases in which decision-making was once again, and by admission of the authors, left to a variety of assessment methods that are neither validated nor consistent. The uncertainty regarding management for this group of women was evident in a paper reporting the recommendations for mode of subsequent delivery from a large UK survey [19]. Although clinical judgement and patient-centred care are always imperative, one of the strengths of our study is that it is the largest study to date to report the outcomes of using a protocol to aid clinicians in recommending mode of subsequent delivery that leaves no “equivocal” group of women. This is important, as it is this very group of women in which clinical practice is likely to vary widely in the absence of a robust evidence base.

It is to this end that we decided to perform a subgroup analysis looking at outcomes for symptomatic women with normal anal manometry and EAUS, who delivered by VD versus CS. This analysis is unique, with other published literature reflecting the guidelines of the RCOG (which by its own admission lack a strong evidence base) recommending that symptomatic women deliver by CS [7]. It is important to remember that although CS broadly speaking prevents further damage to the pelvic floor, it carries a burden of potential morbidity for which women should also be counselled. The CS rate for women included in our study was 19%. This rate is significantly lower than the 50% CS rate of Karmarkar et al., who used the same antenatal assessment methods, but a less detailed protocol [18].

We would also argue that a fourth-degree tear does not preclude vaginal delivery, as has been cautioned in the past [20]. In our cohort, there were 9 fourth-degree index tears. Six of these were recommended CS; 3 were recommended VD (2 asymptomatic with normal anorectal physiology tests and 1 with minor symptoms and normal anorectal physiology tests). We would advocate that referral to a unit able to provide full antenatal assessment with anal manometry and EAUS is essential before making a recommendation on mode of delivery for these women.

We are not aware of any publications that have given consideration to the relevance of IAS defects when recommending mode of delivery. Although the IAS plays a role in maintaining resting pressure, our results show that as long as the EAS is reasonably intact, an IAS defect does not have an impact on bowel symptoms or resting pressure in a subsequent pregnancy. The worsening of squeeze pressure in the VD group at 3 months postpartum for both the intact IAS and IAS defect group (Tables 6, 7, and 8), is consistent with the pattern seen for the whole cohort. We acknowledge that larger numbers are needed to draw firm conclusions, but until that is available it would appear that IAS defects do not predict an adverse outcome during subsequent vaginal delivery.

There is currently a huge variation in the advice given to pregnant women who have sustained a previous OASI and this is reflective of the weak evidence base [19]. A previous national survey showed a wide variation in clinical practice with regard to subsequent mode of delivery recommendations [20]. The RCOG guidelines have changed little from 2007 to 2015, reflecting the fact that the evidence base has not progressed much [7, 8]. A recent survey showed that >50% of respondents did not have a dedicated perineal clinic at their hospitals and did not routinely use symptom questionnaires, anal manometry or EAUS during the follow-up of OASIs [21]. It is a fair assumption that wide-ranging clinical practice still exists.

The strength of this study is in the in-depth information it provides on anorectal symptoms, sphincter function and integrity following subsequent delivery as a result of our rigorous antenatal and postnatal assessment. It therefore has great clinical utility, as we have shown that our framework for managing women in a subsequent pregnancy has resulted in good clinical outcomes for women. We acknowledge that EAUS and anal manometry has financial and workforce implications. However, it has been shown that EAUS has added value as it identifies women in whom OASI has been over-diagnosed and these women can be reassured that they actually sustained a second-degree tear [22]. As a compromise, it may be possible to offer a vaginal delivery to women who are asymptomatic and have good sphincter tone on examination. However, we have previously shown that digital examination has a poor positive predictive value and sphincter injury can be under-diagnosed [23]. Furthermore, at least 10% of women who are asymptomatic in the short term develop anal incontinence by 3 years and this is related to persistent sphincter defects [24]. We advocate that it is imperative for anyone with anorectal symptoms in the absence of an underlying pathological condition such as irritable bowel syndrome to be referred to a centre able to perform full anorectal assessment.

There are limitations to this study. Short-term follow-up is reported and therefore does not provide information on longer-term anorectal symptoms and function. We plan to perform a longer-term follow-up of these women to establish the natural history of subsequent delivery on anorectal symptoms. However, a prospective study by Reid et al., showed no deterioration of anorectal symptoms between 14 weeks postpartum and at 3 years postpartum [24].

Of the 351 women seen in the antenatal period following index OASI, only 169 attended for postnatal follow-up. This is largely due to a number of patients being referred from other centres to our tertiary referral unit. This cohort is still considerably larger than previous studies assessing sphincter integrity, anorectal symptoms and function following a subsequent pregnancy.

Finally, as with all other subsequent pregnancy studies to date, this study is limited by its design. Although a randomised controlled trial would be ideal, in the absence of this option we have aimed to minimise bias as much as possible. This has reduced the potential number we have included in our final analysis, but we aimed to reduce confounding and provide the best evidence that we could within the constraints of a prospective cohort study.

In summary, this study showed that there was no significant worsening of bowel symptoms, anorectal function and sphincter integrity at 12 weeks postpartum, apart from lower squeeze pressures at 3 months postpartum in the VD group among women who complied with the recommended mode of delivery as per our standardised protocol. In the absence of a randomised study, this information and the above protocol will aid clinicians and women in their decision-making regarding subsequent mode of delivery.
